# Cadmium Accumulation and Metallothionein Biosynthesis in Cadmium-Treated Freshwater Mussel *Anodonta woodiana*

**DOI:** 10.1371/journal.pone.0117037

**Published:** 2015-02-03

**Authors:** Yongquan Li, Huizhen Yang, Na Liu, Jixian Luo, Qian Wang, Lan Wang

**Affiliations:** School of Life Science, Shanxi University, Taiyuan, China; University of Naples Federico II, ITALY

## Abstract

This study investigated the distribution of cadmium (Cd) and the protein level of metallothionein (MT) and examined the relationship of Cd accumulation and the MT concentration in different tissues of freshwater mussel *Anodonta woodiana* following Cd treatment. The mussels were exposed to Cd (4.21, 8.43, 16.86, 33.72 and 67.45 mg L^-1^) for 24, 48, 72 and 96 h, respectively. After Cd treatment, the gills, mantle, foot, visceral mass and digestive gland tissues were collected for analysis. We found that, in the controls, Cd distributed in all tissues in the concentration order of gills>mantle>foot>visceral mass>digestive gland. Upon Cd treatment, Cd concentration significantly increased in all tissues. The highest Cd accumulation was found in the digestive gland, which was 0.142 mg g^-1^ (*P*<0.05). MT levels in the gills and mantle of the mussels increased significantly (*P*<0.05), which were in positive correlation with Cd accumulation in the tissues (*P*<0.05). In conclusion, our results demonstrated a correlation between Cd accumulation and MT up-regulation in gills and mantle of the mussels after Cd treatment. It is suggested that the protein level of MT in gills and mantle of *Anodonta woodiana* is a good biomarker for Cd contamination.

## Introduction

Cadmium (Cd) is a biotoxic element and one of the major metals that are ubiquitously distributed in aquatic systems [1–3]. It is also a widely used heavy metal in the industry. In recent years, serious Cd pollution in rivers, estuaries, and near-shore waters has become a serious problem [4]. Anthropogenic input is considered to be the main source of Cd contamination in aquatic environments [5]. Aquatic organisms that encounter waterborne Cd over a long period of time can get adapted and show Cd tolerance [6].

Metallothioneins (MTs) are low molecular weight non-enzymatic proteins that are rich in cysteine, are free of aromatic amino acids and are heat stable. The thiol groups of cysteine residues enable MTs to bind essential and non-essential metals with high affinity [7]. MTs are ubiquitous among mollusc species and play a role in the homeostatic control of essential metals (Cu, Zn) to fulfill enzymatic and metabolic demands [8]. They also play an important role in the detoxification of non-essential trace metals such as Ag, Cd and Hg, which protects organisms against oxidative stress by scavenging intracellular free radicals [9–12]. MT has been used as a biomarker of metal contamination in various species for pollution biomonitoring [7, 13, 14]. This assists in the protections of potamic, lacustrine, estuarine and coastal structure and function.

Recent field studies have demonstrated that Cd can accumulate in aquatic invertebrates, which can cause an elevation of the intracellular level of MT [7, 15, 16]. In freshwater crab *Sinopotamon henanense*, Cd increases the MT levels in the gills and hepatopancreas in time- and dose-dependent, as well as tissue-specific manner [12]. But, there is no relationship between the total Cd, cytosolic Cd and MT level in the digestive gland of most cephalopods from the Bay of Biscay (France) and the Faroe Islands, suggesting the involvement of other Cd-binding ligands [17]. In *Pyganodon grandis*, limnological factors and Cd field contamination can influence the variations in the concentrations of Cd and MT. The pH, calcium (Ca) and Cd at the sediment–water interface explain 74% of the variability in Cd concentrations in the gills. Ca and Cd together explain 62% of the variation in MT concentrations in the gills [18]. Cd detoxification appears to be reasonably effective in the gills and digestive gland of *Pyganodon grandis*, as judged from the protection of the heat-denaturable protein fraction. However, Cd concentration dose increase in potentially metal-sensitive organelles (i.e. mitochondria) in both organs [19]. Moreover, MT protein levels are clearly related to accumulated Cd concentrations in the soft body of *Pyganodon grandis* [20], which is also noted in the whole soft body of two other freshwater bivalves, *Dreissena polymorpha* and *Corbicula fluminea* [21]. The levels of Cu and Cd thioneins increase during metal exposure (3–4 weeks) in the gills and digestive gland of *Mytilus galloprovincialis*, confirming that MTs play a fundamental role in the accumulation of these metals [22]. In aquatic environments, mussels have been widely used for assessing contamination levels, particularly those caused by heavy metals, because of their high bioconcentrating capacities and sensitivity to contaminants [23–26]. The freshwater mussel *Anodonta woodiana* is a common, inexpensive and economically valuable species. This species is widely distributed in rivers and lakes in China, and it plays an important functional role in fresh water ecosystems [27]. Some studies investigated the metabolism and the activities of different enzymes, attempting to use them as potential biomarkers for contamination [28–30]. However, no study has focused on *in vivo* Cd accumulation and MT expression as well as their relationship in the mussel *Anodonta woodiana*.

The present study investigated Cd accumulation and MT protein levels in various tissues of *Anodonta woodiana*, which include the gills, mantle, foot, digestive gland and visceral mass. The relationship between Cd accumulation and MT levels was also examined.

## Materials and Methods

### Ethics Statement

Our study was permitted by Taiyuan government, Shanxi Province, China. We also confirm that the current study did not involve endangered or protected species.

### Chemicals

Cadmium chloride (CdCl_2_), nitric acid and perchloric acid were obtained from Beijing Chemical Reagent Co., Ltd. (Beijing, China). Bovine hemoglobin was purchased from Sigma Co., (St. Louis, MO).

### Animals and experimental design

*Anodonta woodiana* were collected from the Fen River (37°55′ N, 112°14′ E) of Shanxi Province, China. They were acclimated for 2 weeks in glass aquaria filled with dechlorinated and UV-treated tap water (pH 7.5±0.3, dissolved oxygen 8.0±1 mg L^-1^, temperature 20±2°C) under a regime of 12 h light/12 h dark before the experiments. Water was changed every three days. The mussels were fed with commercial feeds every other day.

### Experimental treatment and sample preparation

After acclimation, adult mussels (48.0±4.0 g weight, 6.8±0.3 cm in length) were randomly divided into six groups, with 20 mussels for each group in glass aquaria kept at the same conditions as in the acclimation period, one of which was used as control group. The mussels in other groups were treated with five sub-lethal concentrations of Cd (1/32, 1/16, 1/8, 1/4 and 1/2 of the 96 h LC_50_; the 96 h LC_50_ of acute Cd poisoning is 134.9 mg L^-1^) [30], which were equivalent to 4.21, 8.43, 16.86, 33.72 and 67.45 mg L^-1^ of Cd, respectively. During the experiments, mussels were not fed. Mortality in the treated groups was (10±5%), which was similar to that of the controls. Mussels were treated by Cd under controlled laboratory conditions.

Mussels from the control and treatment groups were collected after 24, 48, 72 and 96 h, respectively. They were depurated in aquarium water for three times and sacrificed on ice. The gills, mantle, foot, digestive gland and visceral mass tissues were dissected, frozen immediately in liquid nitrogen and stored at—80°C until use. Each tissue sample was divided into two parts that were used for Cd and MT analyses, respectively.

### Determination of Cd and MT concentration

To estimate the amount of Cd, the gills, mantle, foot, digestive gland and visceral mass (including gonad and intestine) tissues were digested in 20 ml nitric acid for 12 h at 20°C, then in nitric acid (10 ml) plus perchloric acid (2 ml) for 3 h at 105°C. The concentration of Cd was determined using atomic absorption spectrophotometry (Varian AA240, America) at 228.8 nm according to the method described by Amiard [31], which was expressed as mg g^-1^ wet weight.

The level of MT was determined using the method described by Onosaka [32]. Tissue samples were weighed and homogenized with an ultraturrax homogenizer in Tris-HCl buffer (0.01 M, pH 8.6) at a ratio of 1:9 w/v on ice. The homogenates were then centrifuged at 15000 g for 20 min at 4°C, and aliquots of the supernatant (400 μl) were incubated with 400 μl of CdCl_2_ solution (20 mg L^-1^) at room temperature for 10 min to saturate the metal binding sites of MT. Following an addition of bovine hemoglobin (2%, 200 μl), samples were incubated on ice for 5 min at 4°C and then heat-treated in a water bath for 2 min at 100°C. MT was heat stable and other denatured proteins were removed by centrifugation at 12000 g for 10 min at 4°C. Bovine hemoglobin addition, heat treatment and centrifugation were repeated three times. Cd concentrations were measured in the supernatant by atomic absorption spectrophotometry (Varian AA240, America). Data were expressed as μg g^-1^ wet weight.

### Statistical analysis

Data were presented as mean ± S.D. Multi-group comparison was performed with one-way analysis of variance (ANOVA) using the computer software package SPSS 15.0. Differences among individual groups were determined using the least significant difference test. Pearson correlation coefficient and linear regression analysis were calculated between Cd concentrations and MT levels. *P*<0.05 was considered as statistical significant.

## Results

### Cd accumulation in the tissues of the mussels

**Fig 1:**
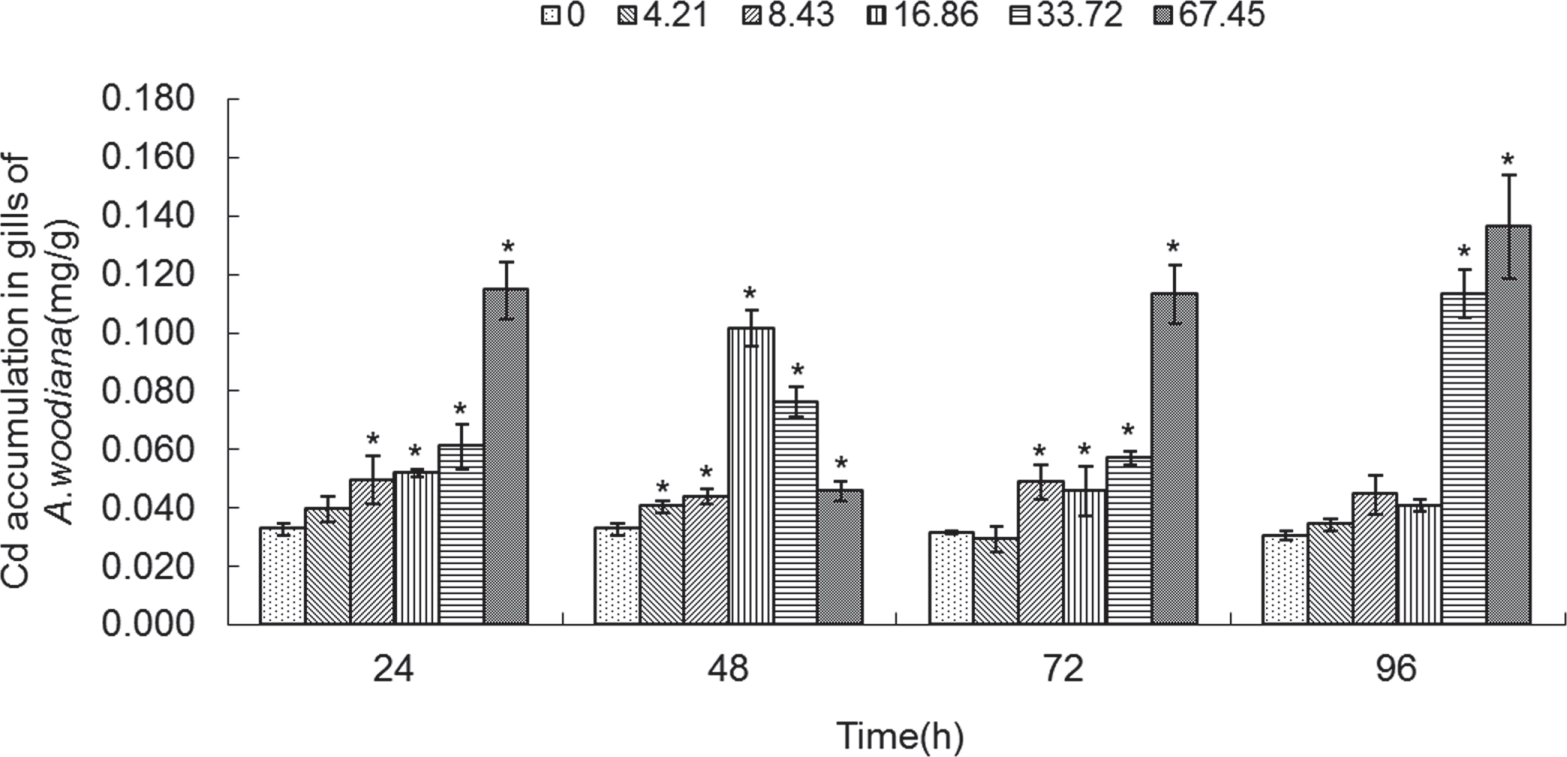
Cd accumulation in gills of *Anodonta woodiana*. Data are expressed as mean ± standard deviation (n = 3). Comparison between the control and treatment groups is notified as * *p <* 0.05.

**Fig 2:**
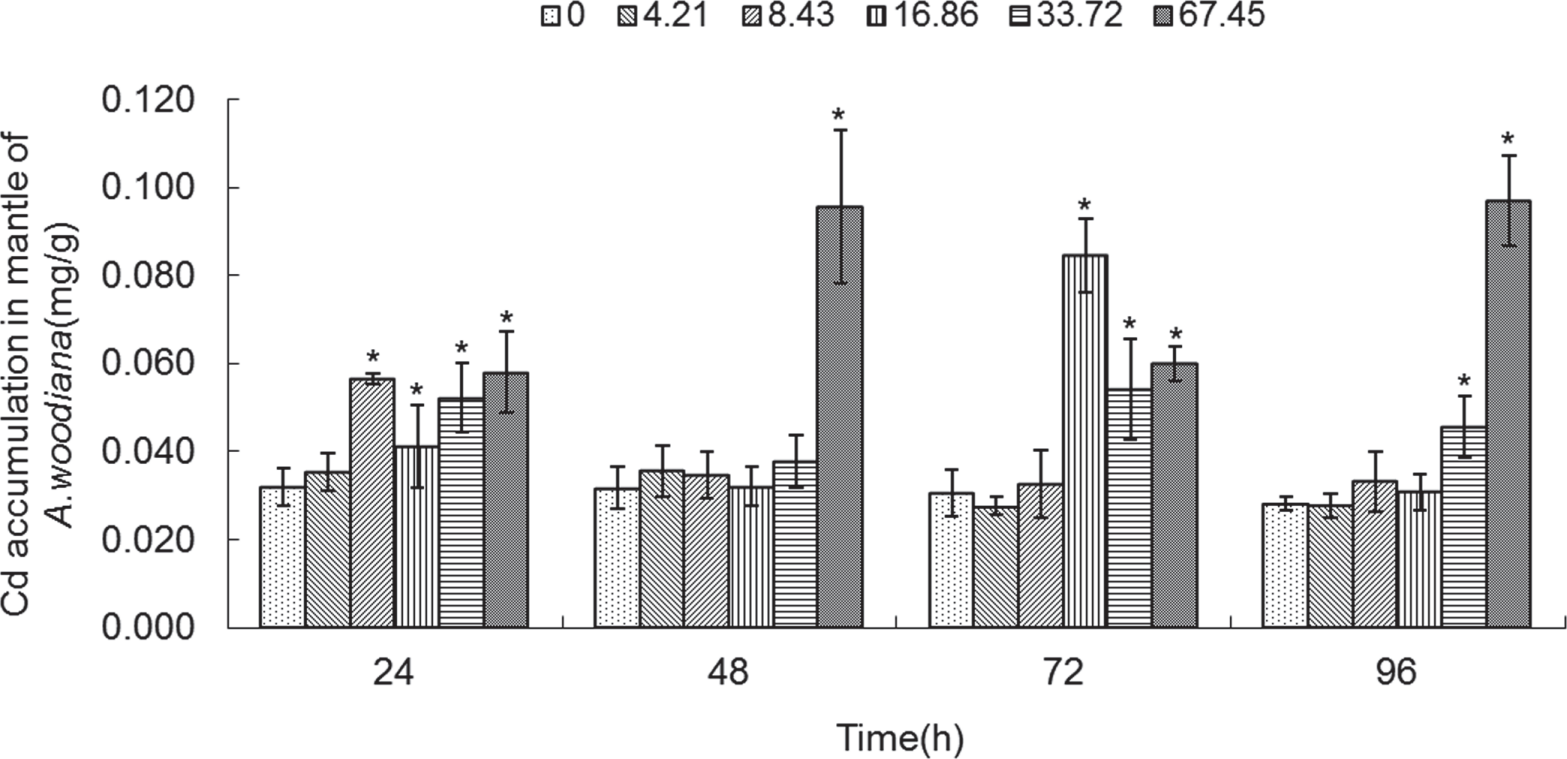
Cd accumulation in mantle of *Anodonta woodiana*. Data are expressed as mean ± standard deviation (n = 3). Comparison between the control and treatment groups is notified as * *p <* 0.05.

**Fig 3:**
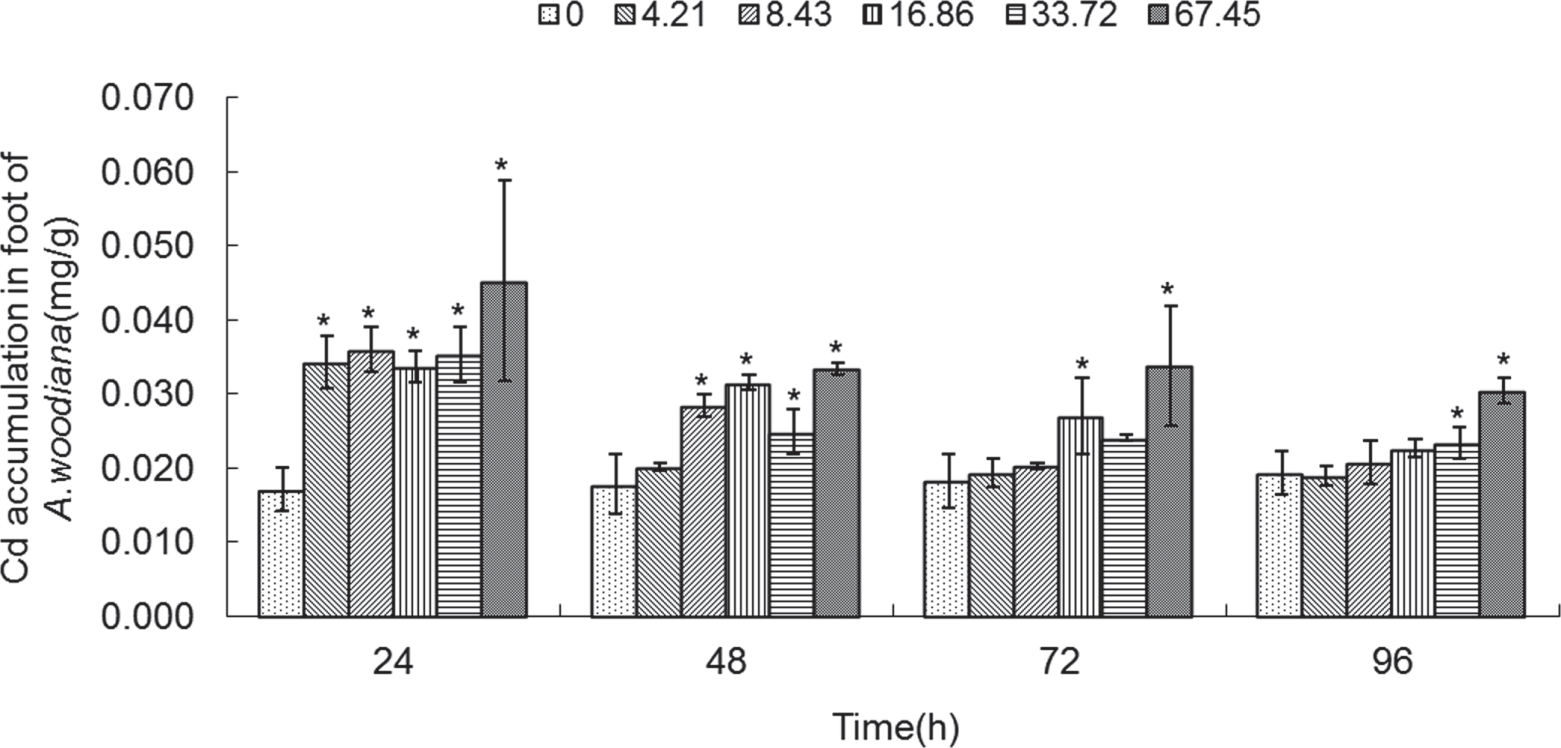
Cd accumulation in foot of *Anodonta woodiana*. Data are expressed as mean ± standard deviation (n = 3). Comparison between the control and treatment groups is notified as * *p <* 0.05.

**Fig 4:**
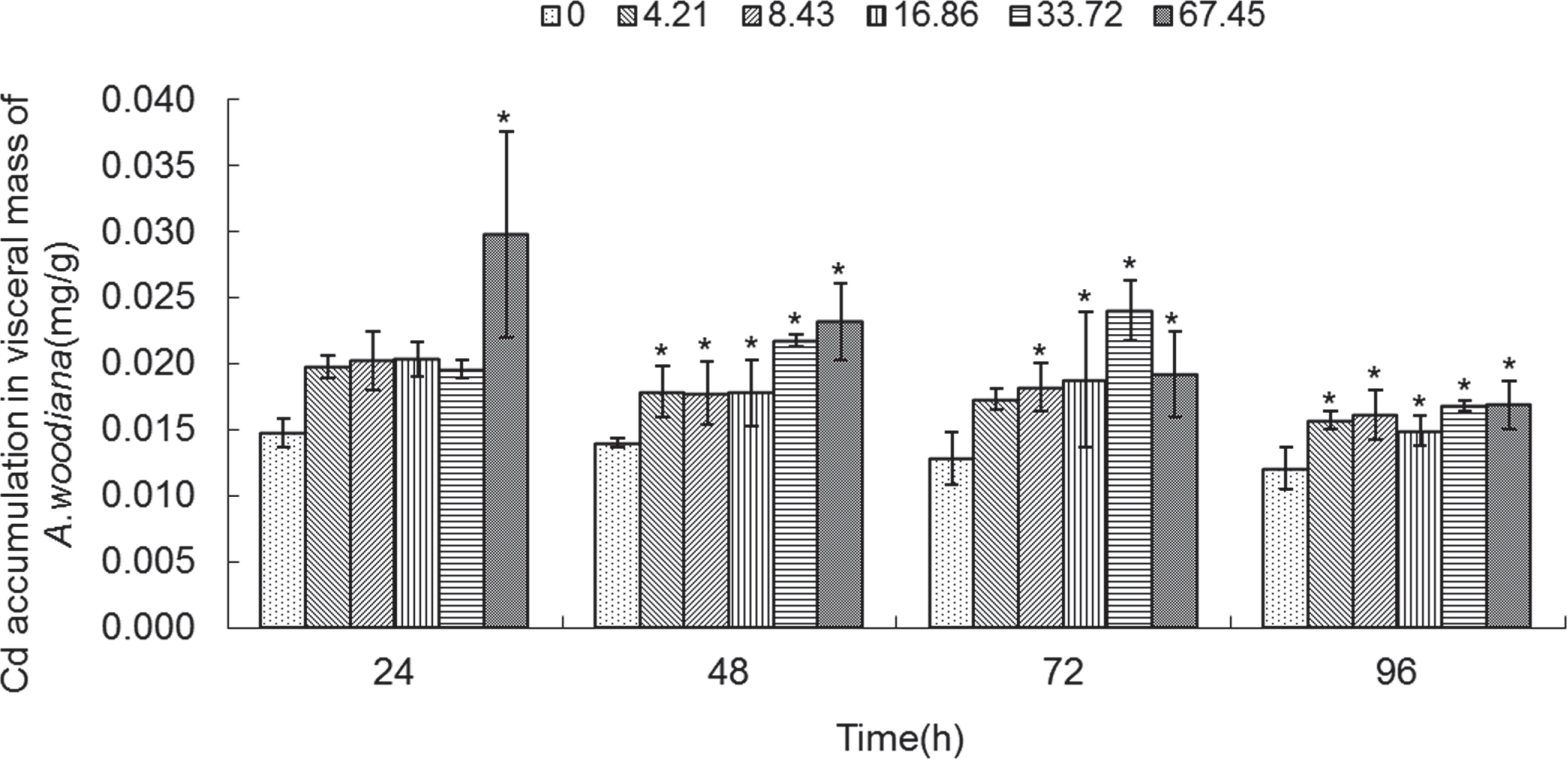
Cd accumulation in visceral mass of *Anodonta woodiana*. Data are expressed as mean ± standard deviation (n = 3). Comparison between the control and treatment groups is notified as * *p <* 0.05.

**Fig 5:**
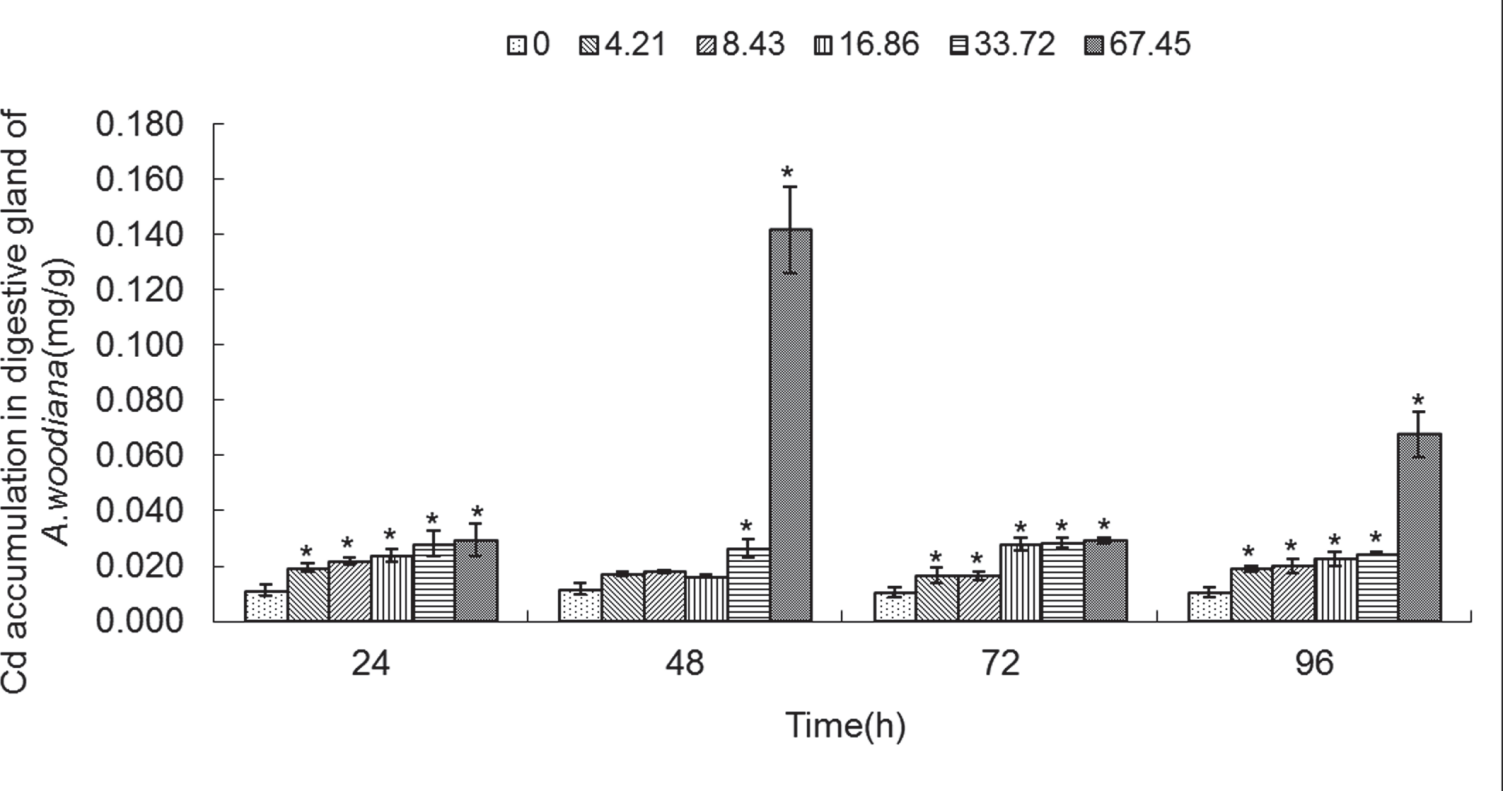
Cd accumulation in digestive gland of *Anodonta woodiana*. Data are expressed as mean ± standard deviation (n = 3). Comparison between the control and treatment groups is notified as * *p <* 0.05.

As shown in **Figs. 1**–**5**, Cd accumulation was measured in different tissues of the mussels. In the controls, Cd was detected in different tissues in the following concentration order: gills>mantle>foot>visceral mass>digestive gland.

In the experimental groups, Cd concentration increased markedly in the gills of the mussels treated with Cd at 8.43, 16.86, 33.72 and 67.45 mg L^-1^ Cd for 24 and 72 h (*P*<0.05). Cd concentration increased in all experimental groups treated with Cd for 48 h (*P*<0.05). At 96 h, it increased to the highest level in the gills treated with Cd (67.45 mg L^-1^) (*P*<0.05, **Fig. 1**). The Cd concentration in mantle of the mussels increased significantly (*P*<0.05) in the following four groups: a. 8.43, 16.86, 33.72 and 67.45 mg L^-1^ Cd for 24 h; b. 67.45 mg L^-1^ Cd for 48 h; c. 16.86, 33.72 and 67.45 mg L^-1^ Cd for 72 h; and d. 33.72 and 67.45 mg L^-1^ Cd for 96 h, which peaked at 67.45 mg L^-1^ Cd for 96 h (*P*<0.05, **Fig. 2**). As shown in **Fig. 3**, when the mussels were exposed to Cd for 24 h, the Cd concentration in foot increased significantly at all of the experimental groups, which peaked in the group of 67.45 mg L^-1^ Cd (all at *P*<0.05). At 48 h, Cd concentration increased notably in the groups treated with Cd at 8.43, 16.86, 33.72 and 67.45 mg L^-1^ (*P*<0.05). At 72 h, it increased notably in the 16.86 and 67.45 mg L^-1^ Cd groups (*P*<0.05). At 96 h, Cd concentration increased in the 33.72 and 67.45 mg L^-1^ Cd groups (*P*<0.05, **Fig. 3**). **Fig. 4** summarized the Cd concentrations in the visceral mass in all experimental groups. At 24 h, the Cd concentration remained unchanged in all experimental groups, except that it increased in the 67.45 mg L^-1^ Cd group (*P*<0.05). Cd concentration increased significantly (all at *P*<0.05) in all other experimental groups of different treatment concentrations and different exposure time points, except the 4.21 mg L^-1^ Cd 72 h group (**Fig. 4**). In the digestive gland, the Cd concentration increased significantly in all of the experimental groups (*P*<0.05), except the three 48 h groups (4.21, 8.43, 16.86 mg L^-1^, respectively), and it reached the highest level in the 67.45 mg L^-1^ Cd 48 h group (**Fig. 5**).

### MT concentration in tissues of mussels treated with Cd

**Fig 6:**
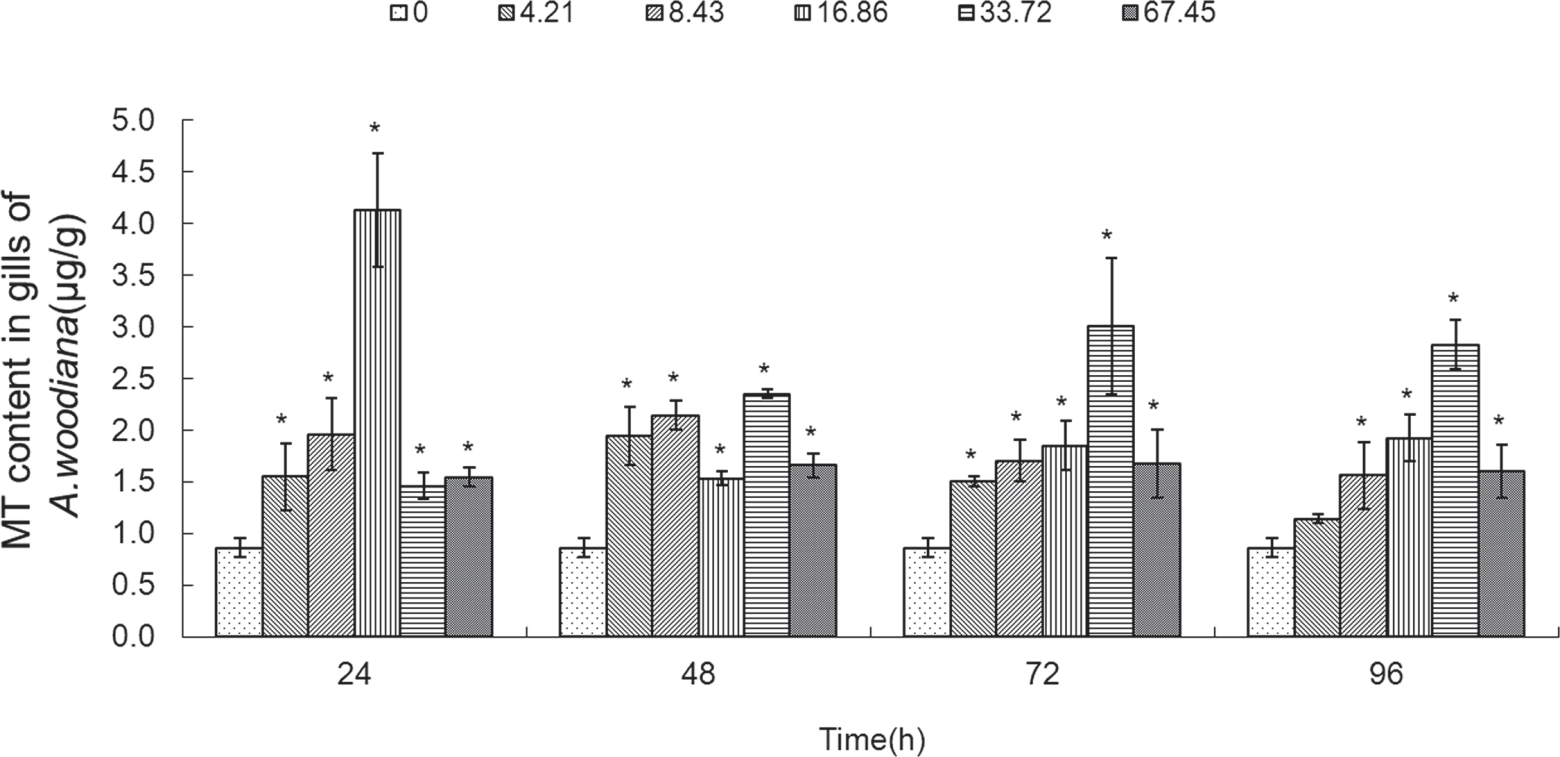
MT content in gills of *Anodonta woodiana*. Data are expressed as mean ± standard deviation (n = 3). Comparison between the control and treatment groups is notified as * *p <* 0.05.

**Fig 7:**
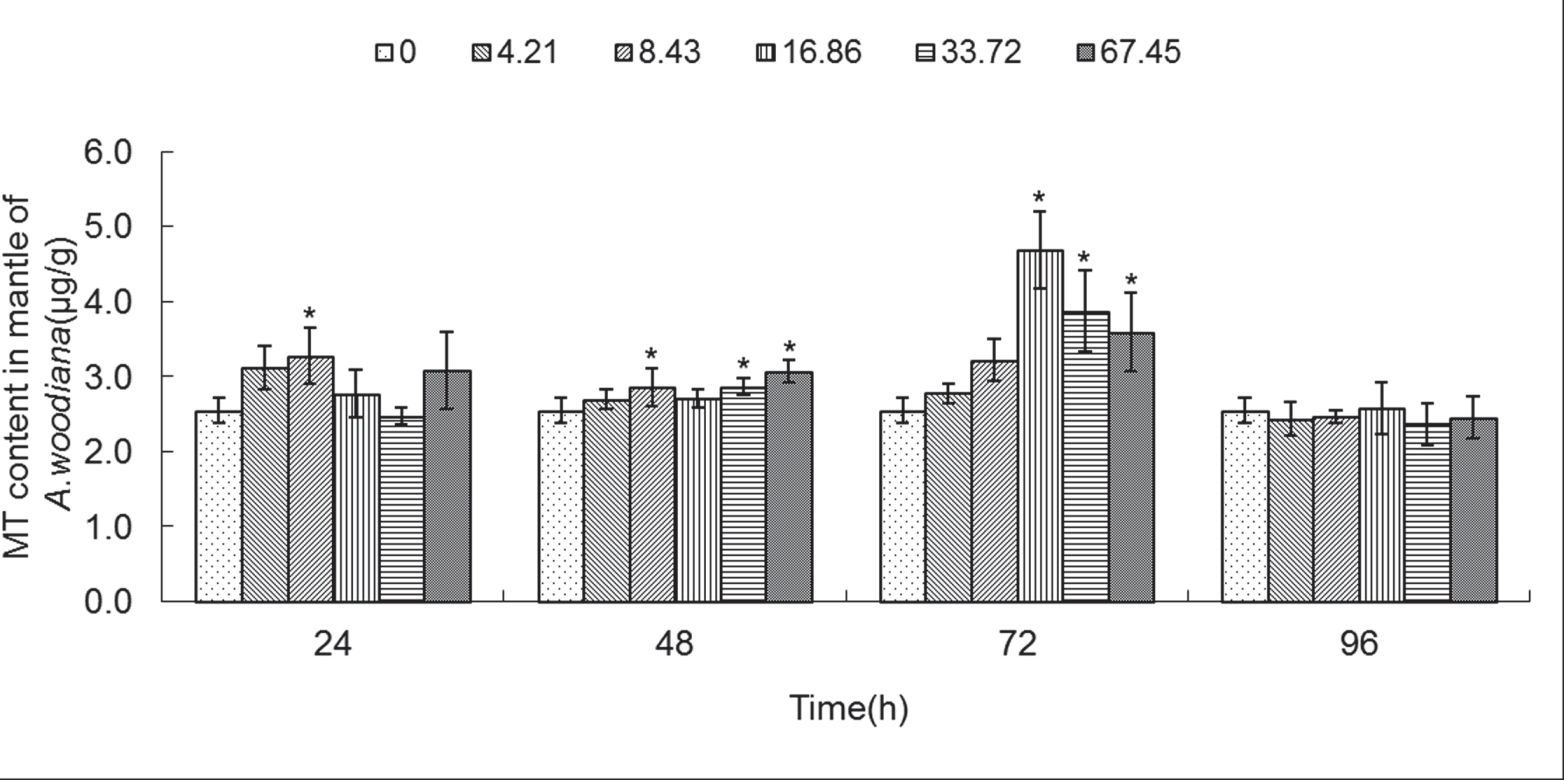
MT content in mantle of *Anodonta woodiana*. Data are expressed as mean ± standard deviation (n = 3). Comparison between the control and treatment groups is notified as * *p <* 0.05.

**Fig 8:**
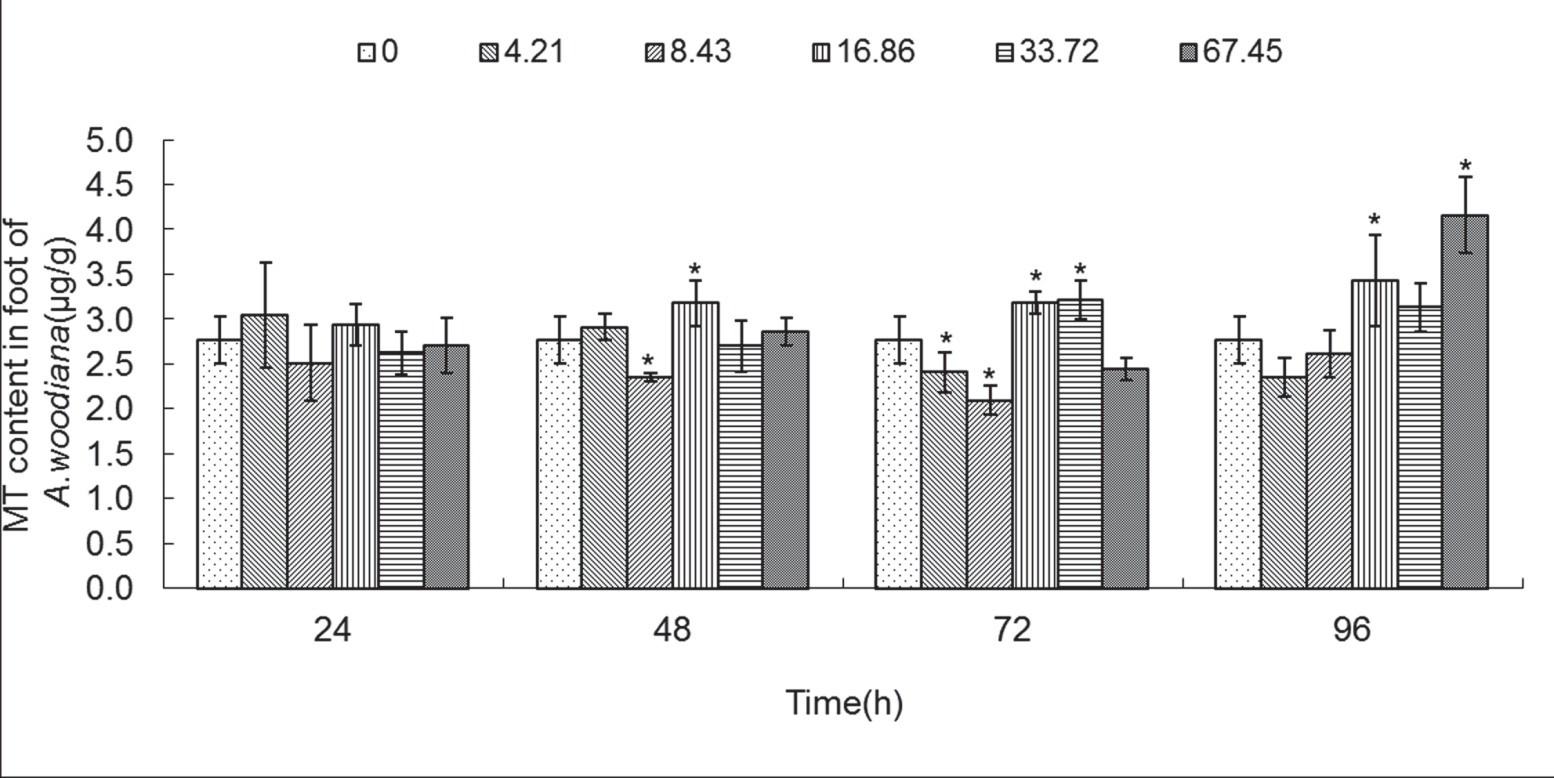
MT content in foot of *Anodonta woodiana*. Data are expressed as mean ± standard deviation (n = 3). Comparison between the control and treatment groups is notified as * *p <* 0.05.

**Fig 9:**
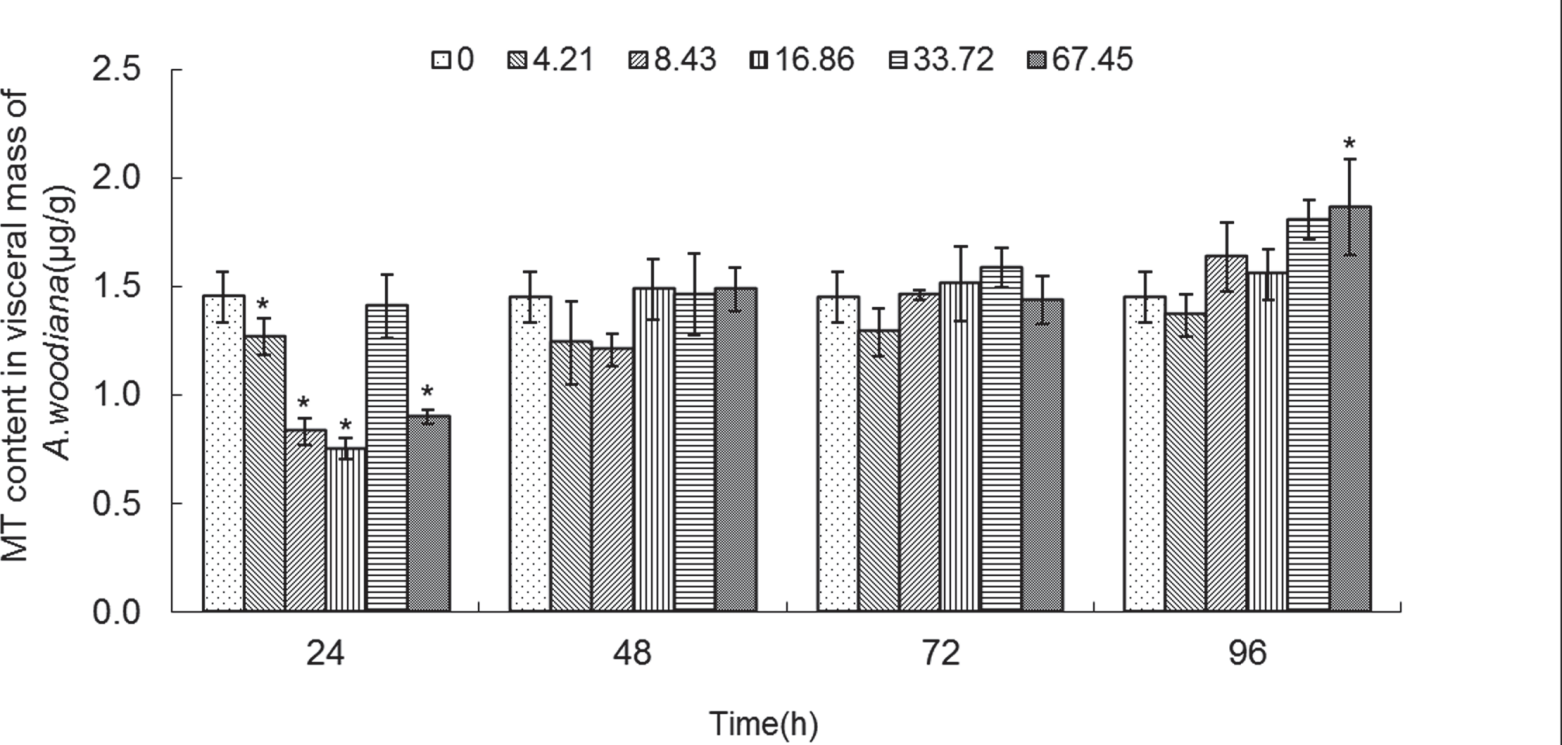
MT content in visceral mass of *Anodonta woodiana*. Data are expressed as mean ± standard deviation (n = 3). Comparison between the control and treatment groups is notified as * *p <* 0.05.

**Fig 10:**
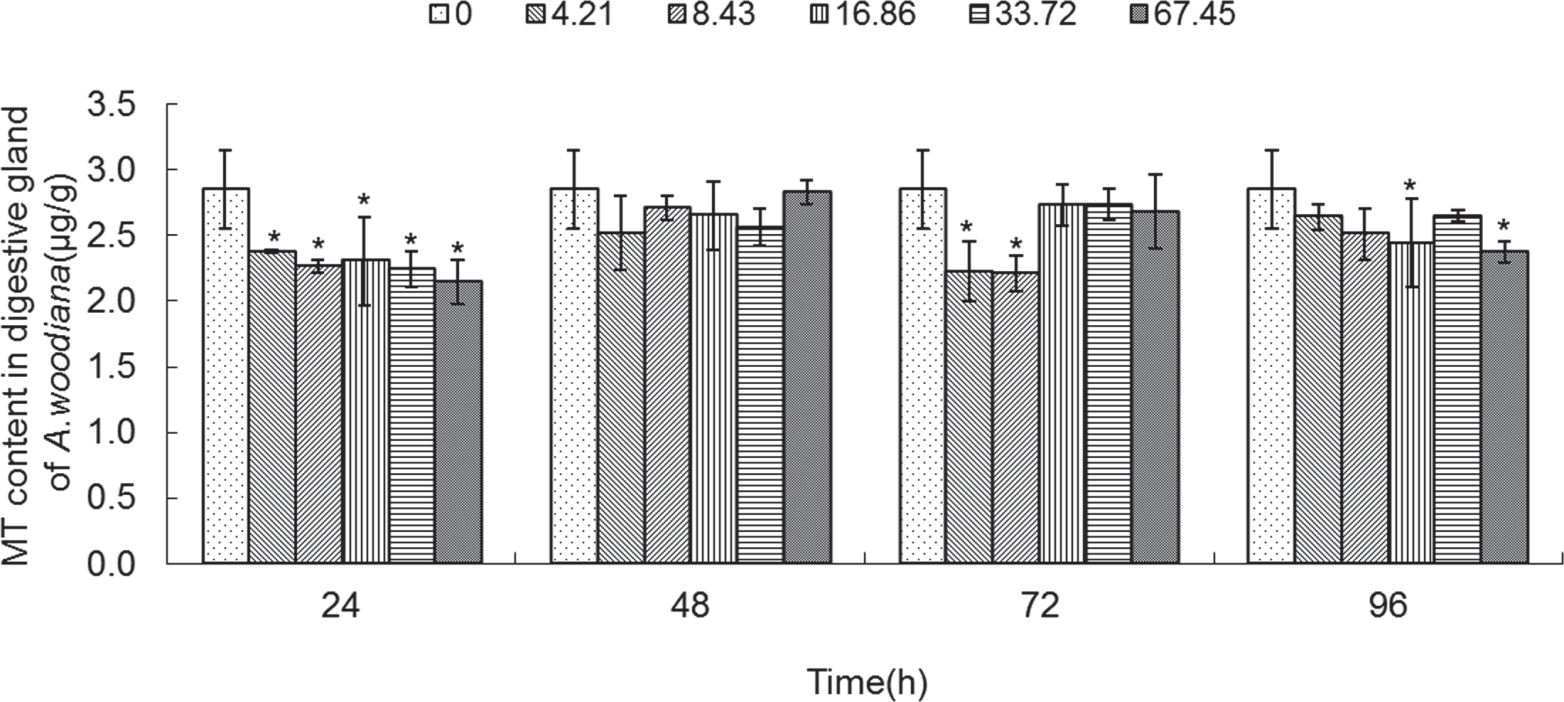
MT content in digestive gland of *Anodonta woodiana*. Data are expressed as mean ± standard deviation (n = 3). Comparison between the control and treatment groups is notified as * *p <* 0.05.

MT levels in different tissues of the mussels exposed to Cd were shown in **Figs. 6**–**10**. In the controls, the order of MT content in different tissues was digestive gland>foot>mantle>visceral mass>gills.

As shown in **Fig. 6**, the MT level increased in gills in all of the experimental groups (*P*<0.05), except the 4.21 mg L^-1^ Cd 96 h group. MT level reached a maximum in the 16.86 mg L^-1^ Cd 24 h group (**Fig. 6**). As shown in **Fig. 7**, the MT level in mantle tissue increased only in the 8.43 mg L^-1^ Cd group for 24 h. At 48 h, the MT level increased in the 8.43, 33.72 and 67.45 mg L^-1^ Cd groups. At 72 h, the MT level increased in the 16.86, 33.72 and 67.45 mg L^-1^ Cd groups, which reached the highest level in the 16.86 mg L^-1^ Cd group. It remained unchanged in all the experimental groups for 96 h (**Fig. 7**). In the foot (**Fig. 8**), the MT level decreased in the 8.43 mg L^-1^ Cd group, but increased in the 16.86 mg L^-1^ Cd group for 48 h (*P*<0.05). At 72 h, the MT protein concentration decreased significantly in the groups treated with lower Cd concentrations (4.21 and 8.43 mg L^-1^), but increased in the groups treated with higher Cd concentrations (16.86 and 33.72 mg L^-1^, *P*<0.05). The MT protein concentration peaked in the 67.45 mg L^-1^ Cd 96 h group (*P*<0.05, **Fig. 8**). In the visceral mass of the mussels exposed to Cd (4.21, 8.43, 16.86 and 67.45 mg L^-1^) for 24 h, the MT level decreased significantly (*P*<0.05) and it remained unchanged for 48 and 72 h. In contrast, it increased notably in the 67.45 mg L^-1^ 96 h group (**Fig. 9**). As shown in **Fig. 10**, the MT level in the digestive gland decreased at 24 h in all of the treatment groups (*P*<0.05). A decrease in MT level was also noted in some other experimental groups which included 4.21 and 8.43 mg L^-1^ groups (72 h) as well as 16.86 and 67.45 mg L^-1^ groups (96 h). The MT level remained unchanged in the experimental groups treated for 48 h (**Fig. 10**).

### Correlations between Cd accumulation and MT concentration

In order to examine the relationship between Cd accumulation and the protein level of MT, we performed statistical analyses using the Pearson correlation coefficient and linear regression analysis. As shown in **Table 1**, in the gills, Cd treatment markedly increased the MT levels in a concentration-dependent manner (96 h, *P*<0.05), and the tissue Cd accumulation correlated positively with the protein level of MT (*P*<0.05). Similarly, a positive correlation between Cd concentration and the protein level of MT was found in the mantle (48h and 72h, *P*<0.05). Surprisingly, the MT protein concentration in the digestive gland showed a negative correlation with Cd accumulation (24 h, *P*<0.05).

**Table 1:** Correlations between Cd accumulation and MT levels.

Tissue	Time (h)	Correlation coefficients	Regression equation	P value. (2-tailed)
Gills	96	0.568	y = 8.767x+1.066	0.014
Mantle	48	0.628	y = 5.598x+2.542	0.005
	72	0.815	y = 30.504x+1.971	0.000
Digestive gland	24	−0.665	y = -28.077x+2.991	0.003

## Discussion

Assessing the biological effects of pollutants during biomonitoring attempts show that biomarkers are of high demand for the biomonitoring in aquatic ecosystems [33]. Ecotoxicological programs recommend the use of even a set of biomarkers for ecosystems health and environmental risk assessments [34].

MTs are generally considered as useful biomarkers for heavy metal pollution, because their protein concentration often increases upon exposure to heavy metals in different organs [35]. When bivalves *Mytilus edulis* and *Mytilus galloprovincialis* are exposed to Cd, a linear relationship between Cd and MT concentrations is observed in the whole soft tissues [36, 37]. In the oysters *Crassostrea gigas*, the MT levels in the digestive gland are only occasionally correlated with accumulated metal concentrations [38].

Although metal induction of MT synthesis is often observed, some studies do not show a positive correlation between heavy metal accumulation and MT biosynthesis in invertebrates. In the clam *Ruditapes decussatus* exposed to Cd (400 μg L^-1^) for 30 days, MT has no significant increase in the soft tissues [39]. On the other hand, a significant increase in MT10 appears only after 9 days of exposure to Cd in the digestive gland of *Mytilus galloprovincialis*. In contrast, the protein concentration of MT10 is significantly reduced after 2 days exposure to Cu [40], suggesting that MT expression in the digestive gland is different in response to different heavy metals. Some studies even show the negative relationships between Cd and MT concentrations. In the copepods *Tigriopus brevicornis*, Cd can decrease the concentrations of MTs [41]. Thus, the relationship between heavy metal contamination and the increase in MT concentration is controversial. At sites where metals are present at high concentrations, some species do not show increased MT concentrations, at least in some organs [38, 42–44]. Further studies are required to clarify the relationship between metal contamination and MT level and to validate the use of MTs as biomarkers [7].

In the present work, we found that Cd accumulation provoked a significant increase of MT protein concentration in the gills and mantle of *Anodonta woodiana* (**Fig. 1; Fig. 2; Fig. 6; Fig. 7; Table 1**). In contrast, there was no correlation between Cd accumulation and MT protein level in the foot and visceral mass. This supports the view of a positive correlation between Cd and MT in specific tissues [12, 40, 45]. Mussels filter large volumes of water through their gills and mantle, and thus concentrate contaminants in these tissues. Gills and mantle are the main interface between the organism and its environment, and hence these tissues are frequent targets of environmental pollutants. Any adverse change in the ambient environment, such as oxidative stress, is easily reflected in these tissues [46, 47]. In the digestive gland, we even found a negative correlation between Cd accumulation and MT protein level (**Fig. 5**; **Fig. 10**; **Table 1**), suggesting that MT did not play a main role in the detoxification or homeostasis of Cd in the tissue. Cadmium detoxification can occur with other metalloproteins rather than MTs. The digestive gland consists of numerous blind ending tubules that are composed of digestive and basophilic cells. The digestive gland is involved in digestive and absorptive functions, and its epithelium is composed of cells rich in lysosomes which contain several hydrolytic enzymes [17]. In invertebrates, similar to bivalves, lysosomes are known to accumulate both essential and toxic metals from the cytosol of the digestive gland cells, and play an important role in Cd detoxification [17, 48]. After the freshwater mussel *Dreissena polymorpha* are treated with 200 μg L^-1^ of Cd for 7 days, digestive lysosomal system is activated, as evidenced by an increase in both the number and the size of lysosomes [34]. So, it is not surprising that metals are found in lysosomal residual bodies [49]. Lysosomal system has several typical structures, i.e. heterolysosomes and heterophagosomes, ‘‘boules”, residual bodies and brown bodies. Such lysosomal structures are probably involved in the compartmentalisation of metals in the digestive gland cells [17, 50]. It appears that the effect of Cd on MTs protein level is tissue-specific. Our data suggest that Cd and MT levels in the gills and mantle of *Anodonta woodiana* provide good indicators for Cd pollution, although, MT levels in the digestive gland have been widely used as biomarkers for pollution by metals including Cd [13, 51–54].

The mechanisms of MT gene expression are poorly characterized as yet, particularly during oxidative stress. The Zn-sensitive inhibitor regulates MT gene expression, which provides a conceptual model for interpreting the significance of MT induction in response to toxic metals [55]. In this model, Cd has greater ligand affinity than Zn and it can displace Zn from other Zn binding sites via metal-metal exchange reaction. The displaced Zn is then available for binding the metal transcription inhibitor (MTI), releasing the metal transcriptional factor (MTF) from inhibition, and initiating MT expression [8]. The presence of metal regulatory elements (MREs) in the upstream sequences of MT gene is indicative of the specificity of this induction. Studies indicate that MREs play a positive role in initiating MT gene expression [56]. In mice, MT gene expression is regulated by Zn-mediated release of an inhibitor. The six zinc-finger metal-responsive transcription factor MTF-1 plays a central role in the transcriptional activation of the MT-I gene in response to zinc. MTF-1 is induced to bind to the metal response elements in the proximal MT promoter in cells during oxidative stress [10]. The basic helix-loop-helix-leucine zipper protein, upstream stimulatory factor family (USF), also plays a role in regulating the transcription of the mouse MT-I gene in response to Cd or H_2_O_2_ [57–59]. Studies also demonstrate that cis-acting elements mediate the induction of MT gene expression by metals and oxidative stress, which is present in the chicken MT promoter. This suggests a role for increased binding of the transcription factor MTF-1 to the metal response elements [60]. Whether MTF-1- or USF-like protein functions in the freshwater mussel *Anodonta woodiana* in response to metals has not yet been investigated. Our results showed that Cd increased the MT biosynthesis in the freshwater mussel *Anodonta woodiana*, which may be mediated by the transcription factor of MTF-1- or USF-like protein.

We have evidence that H_2_O_2_ accumulated in various tissues of freshwater mussel *Anodonta woodiana* after Cd treatment (data not shown), indicating that Cd triggers ROS production. ROS can induce protein oxidation, especially targeting the proteins rich in cysteines. MTs are low molecular weight proteins rich in cysteines. Within a total of 61–62 amino acids, depending on their source, there are 32 cysteines [61]. In our experiment, we found an increase in MT in gills and mantle, suggesting that the increase in MT maybe one of the responses to Cd, which is to reduce the Cd-induced ROS. From this point of view, a higher level of MT is beneficial, which can reduce oxidative damages induced by Cd.

MTs play important roles in the detoxification of heavy metals including Cd and Hg by binding with heavy metals through the thiol group of cysteine residues [62, 63]. Therefore, an increase in MT mRNA/protein levels may reflect an elevated demand to detoxify Cd. This is the reason why MTs have been widely used as significant biomarkers for metal pollution. However, it is worth noting that no increase in MT proteins was observed in organs of fish exposed to metal, despite that the MT gene transcription was up-regulated. This suggests that the physiological role of MT gene transcription can be increased in response to MT degradation by metal, attempting by cells to maintain MT levels; increase in transcription alone does not increase metal sequestration capacities of MT [64].

## Conclusions

In conclusion, our results showed that Cd accumulated in all tissues to different levels. Concomitantly, there was an increase in protein levels of MT only in gills and mantle. Positive correlations between Cd and MT levels were found in these two tissues (**Table 1**). Our results indicate that positive correlation between Cd and MT is tissue-specific, and suggest that protein level of MT in gills and mantle of *Anodonta woodiana* is a useful biomarker for Cd pollution.

## References

[1] Novelli F, Novelli E, Manzano MA, Lopes AM, Cataneo AC, et al (2000) Effect of tocopherol on superoxide radical and toxicity of cadmium exposure. Int J Environ Health Res 10: 125–134.

[2] Campbell PGC (2006) Cadmium-a priority pollutant. Environ Chem 3: 387–388.

[3] Burger J (2008) Assessment and management of risk to wildlife from cadmium. Sci Total Environ 389: 37–45. 17910979 10.1016/j.scitotenv.2007.08.037

[4] Fingerman M, Devi M, Reddy PS, Katyayani R (1996) Impact of heavy metal exposure on the nervous system and endocrine mediated processes in Crustaceans. Zool Stud 35: 1–8.

[5] Kalman J, Riba I, DelValls T, Blasco J (2010) Comparative toxicity of cadmium in the commercial fish species *Sparus aurata* and *Solea senegalensis* . Ecotoxicol Environ Saf 73: 306–311. 10.1016/j.ecoenv.2009.10.013 19913912

[6] Silvestre F, Duchêne C, Trausch G, Devos P (2005) Tissue-specific cadmium accumulation and metallothionein-like protein levels during the acclimation process in the Chinese crab *Eriocheir sinensis* . Comp Biochem Physiol C 140: 39–45. 15792621 10.1016/j.cca.2005.01.004

[7] Amiard JC, Amiard-Triquet C, Barka S, Pellerin J, Rainbow PS (2006) Metallothioneins in aquatic invertebrates: Their role in metal detoxification and their use as biomarkers. Aquat Toxicol 76: 160–202. 16289342 10.1016/j.aquatox.2005.08.015

[8] Roesijadi G (1996) Metallothionein and its role in toxic metal regulation. Comp Biochem Physiol C 113: 117–123.

[9] Langston WJ, Bebianno MJ, Burt GR (1998) Metal handling strategies in molluscs. In: Langston WJ, Bebianno MJ (Eds.), Metal Metabolism in Aquatic Environments. Chapman and Hall, London, pp. 219–283.

[10] Andrews GK (2000) Regulation of metallothionein gene expression by oxidative stress and metal ions. Biochem Pharmacol 59: 95–104. 10605938 10.1016/s0006-2952(99)00301-9

[11] Mao H, Wang DH, Yang WX (2012) The involvement of metallothionein in the development of aquatic invertebrates. Aquat Toxicol 110–111: 208–213. 10.1016/j.aquatox.2012.01.018 22343466

[12] Li Y, Chai X, Wu H, Jing W, Wang L (2013) The response of metallothionein and malondialdehyde after exclusive and combined Cd / Zn exposure in the crab *Sinopotamon henanense* . PloS ONE 8(11): e80475 10.1371/journal.pone.0080475 24260400 PMC3832363

[13] Zorita I, Strogyloudi E, Buxens A, Mazón LI, Papathanassiou E, et al (2005) Application of two SH-based methods for metallothionein determination in mussels and intercalibration of the spectrophotometric method: laboratory and field studies in the Mediterranean Sea. Biomarkers 10: 342–359. 16243720 10.1080/13547500500264645

[14] Ladhar-Chaabouni R, Machreki-Ajmi M, Hamza-Chaffai A (2012) Use of metallothioneins as biomarkers for environmental quality assessment in the Gulf of Gabès (Tunisia). Environ Monit Assess 184: 2177–2192. 10.1007/s10661-011-2108-5 21611846

[15] Ma WL, Wang L, He YJ, Yan T (2008) Tissue-specific cadmium and metallothionein levels in freshwater crab *Sinopotamon henanense* during acute exposure to waterborne cadmium. Environ Toxicol 23: 393–400. 10.1002/tox.20339 18214890

[16] Ma WL, Yan T, He YJ, Wang L (2009) Purification and cDNA Cloning of a Cadmium-Binding Metallothionein from the Freshwater Crab *Sinopotamon henanense* . Arch Environ Contam Toxicol 56: 747–753. 10.1007/s00244-008-9224-4 18846314

[17] Bustamante P, Cosson RP, Gallien I, Caurant F, Miramand P (2002) Cadmium detoxification processes in the digestive gland of cephalopods in relation to accumulated cadmium concentrations. Mar Environ Res 53: 227–241. 11939291 10.1016/s0141-1136(01)00108-8

[18] Perceval O, Pinel-Alloul B, Méthot G, Couillard Y, Giguère A, et al (2002) Cadmium accumulation and metallothionein synthesis in freshwater bivalves (*Pyganodon grandis*): relative influence of the metal exposure gradient versus limnological variability. Environ Pollut 118: 5–17. 11996382 10.1016/s0269-7491(01)00282-2

[19] Campbell PG, Giguère A, Bonneris E, Hare L (2005) Cadmium-handling strategies in two chronically exposed indigenous freshwater organisms–the yellow perch (*Perca flavescens*) and the floater mollusc (*Pyganodon grandis*). Aquat Toxicol 72: 83–97. 15748749 10.1016/j.aquatox.2004.11.023

[20] Couillard Y, Campbell PG, Tessier A (1993) Response of metallothionein concentrations in a freshwater bivalve *Anodonta grandis* along an environmental cadmium gradient. Limnol Oceanogr 38: 299–313.

[21] Marie V, Gonzalez P, Baudrimont M, Bourdineaud JP, Boudou A (2006) Metallothionein response to cadmium and zinc exposures compared in two freshwater bivalves, *Dreissena polymorpha* and *Corbicula fluminea* . Biometals 19: 339–407. 10.1007/s10534-005-4064-4 16841249

[22] Viarengo A, Palmero S, Zanicchi G, Capelli R, Vaissiere R, et al (1985) Role of metallothioneins in Cu and Cd accumulation and elimination in the gill and digestive gland cells of *Mytilus galloprovincialis* lam. Mar Environ Res 16: 23–36.

[23] Boening DW (1999) An evaluation of mussels as biomonitors of heavy metal pollution in marine waters. Environ Monit Assess 55: 459–470.

[24] Blaise C, Gagné F, Pellerin J, Hansen PD, Trottier S (2002) Molluscan shellfish biomarker study of the Québec, Canada, Saguenay Fjord with the soft-shell clam *Mya arenaria* . Environ Toxicol 17: 170–186. 12112625 10.1002/tox.10048

[25] Zhou QF, Li ZY, Jiang GB, Yang RQ (2003) Preliminary investigation of a sensitive biomarker of organotin pollution in Chinese coastal aquatic environment and marine organisms. Environ Pollut 125: 301–304. 12826407 10.1016/s0269-7491(03)00145-3

[26] Pellerin J, Amiard JC (2009) Comparison of bioaccumulation of metals and induction of metallothioneins in two marine mussels (*Mytilus edulis* and *Mya arenaria*). Comp Biochem Physiol C 150: 186–195. 10.1016/j.cbpc.2009.04.008 19409510

[27] Liu LY, Zheng GM (2009) General Zoology. Higher Education Press, Beijing, pp. 208 10.1016/j.mrgentox.2009.07.012

[28] Corsi I, Pastore AM, Lodde A, Palmerini E, Castagnolo L, et al (2007) Potential role of cholinesterases in the invasive capacity of the freshwater mussel, *Anodonta woodiana* (Bivalvia: Unionacea): A comparative study with the indigenous species of the genus, *Anodonta sp* . Comp Biochem Physiol C 145: 413–419. 17324630 10.1016/j.cbpc.2007.01.011

[29] Kim BH, Lee JH, Hwang SJ (2011) Inter- and intra-specific differences in filtering activities between two unionids, *Anodonta woodiana* and *Unio douglasiae*, in ambient eutrophic lake waters. Ecol Eng 37: 1957–1967.

[30] Xing HF, Li YQ, Yang HZ, Wang L (2013) Effects of cadmium on antioxidant enzyme activities and lipid peroxidation in the mantle and gill of the freshwater mussel *Anodonta woodiana* . Acta Scien Circum 33: 856–860.

[31] Amiard JC, Pineau HL, Boiteau C, Metayer C, Amiard-Triquet C (1987) Application of the Zeeman atomic absorption spectrometry to the measurement of eight trace elements (Ag, Cd, Cr, Cu, Mn, Ni, Pb and Se) in biological matrices solid. Water Res 21: 693–697.

[32] Onosaka S, Cherian MG (1982) Comparison of metallothionein determination by polarographic and cadmium-saturation methods. Toxicol Appl Pharmacol 63: 270–274. 7089976 10.1016/0041-008x(82)90049-7

[33] Clements WH (2000) Integrating effects of contaminants across levels of biological organization: an overview. J Aquat Ecosyst Stress Recovery 7: 113–116.

[34] Giambérini L, Cajaraville MP (2005) Lysosomal responses in the digestive gland of the freshwater mussel, *Dreissena polymorpha*, experimentally exposed to cadmium. Environ Res 98: 210–214. 15820727 10.1016/j.envres.2004.11.003

[35] Wu JP, Chen HC (2005) Metallothionein induction and heavy metal accumulation in white shrimp *Litopenaeus vannamei* exposed to cadmium and zinc. Comp Biochem Physiol C 140: 383–394. 15925547 10.1016/j.cca.2005.03.006

[36] Bebianno MJ, Langston WJ (1991) Metallothionein induction in *Mytilus edulis* exposed to cadmium. Mar Biol 108: 91–96.

[37] Bebianno MJ, Langston WJ (1992) Cadmium induction of metallothionein synthesis in *Mytilus galloprovincialis* . Comp Biochem Physiol C 103: 79–85.

[38] Geffard A, Amiard-triquet C, Amiard JC, Mouneyrac C (2001) Temporal variations of metallothionein and metal concentrations in the digestive gland of oysters *Crassostrea gigas* from a clean and a metal-rich site. Biomarkers 6: 91–107. 10.1080/13547500010000860 23886106

[39] Bebianno MJ, Nott JA, Langston WJ (1993) Cadmium metabolism in the clam *Ruditapes decussata*: the role of metallothioneins. Aquat Toxicol 27: 315–334.

[40] Zorita I, Bilbao E, Schad A, Cancio I, Soto M, et al (2007) Tissue- and cell-specific expression of metallothionein genes in cadmium- and copper-exposed mussels analyzed by in situ hybridization and RT-PCR. Toxicol Appl Pharmacol 220: 186–196. 17350662 10.1016/j.taap.2007.01.003

[41] Barka S, Pavillon JF, Amiard JC (2001) Influence of different essential and non-essential metals on MTLP levels in the copepod *Tigriopus brevicornis* . Comp Biochem Physiol C 128: 479–493. 11301290 10.1016/s1532-0456(00)00198-8

[42] Pedersen SN, Lundebye AK (1996) Metallothionein and stress protein levels in shore crabs (*Carcinus maenas*) along a trace metal gradient in the Fal estuary (UK). Mar Environ Res 42: 241–246.

[43] Géret F, Cosson RP (2000) Utilisation des métallothionéines comme biomarqueur de la contamination métallique: variabilité entre sites et organes chez 1’huître *Crassostrea gigas* . Oceanol Acta 23: 261–271.

[44] Berthet B, Mouneyrac C, Amiard JC, Amiard-Triquet C, Berthelot Y, et al (2003) Response to metals of the polychaete annelid *Hediste diversicolor*, a key species in estuarine and coastal sediments. Arch Environ Contam Toxicol 45: 468–478. 14708663 10.1007/s00244-003-0135-0

[45] Wang J, Zhang P, Shen Q, Wang Q, Liu D, et al (2013) The effects of cadmium exposure on the oxidative state and cell death in the gill of freshwater crab *Sinopotamon henanense* . PLoS ONE 8(5): e64020 10.1371/journal.pone.0064020 23737962 PMC3667791

[46] Rajalakshmi S, Mohandas A (2005) Copper-induced changes in tissue enzyme activity in a freshwater mussel. Ecotoxicol Environ Saf 62:140–143. 15978300 10.1016/j.ecoenv.2005.01.003

[47] Cossu C, Doyotte A, Jacquin MC, Babut M, Exinger A, et al (1997) Glutathione reductase, selenium-dependent glutathione peroxidase, glutathione levels and lipid peroxidation in freshwater bivalves, *Unio tumidus* as biomarkers of aquatic contamination in field studies. Ecotoxicol Environ Saf 38: 122–131. 9417853 10.1006/eesa.1997.1582

[48] Moore MN (1990) Lysosomal cytochemistry in marine environmental monitoring. Histochem J 22: 187–191. 2201669 10.1007/BF02386003

[49] Dallinger R (1995) Mechanisms of metal incorporation into cells. University of the Basque Country Press, Bilbo, pp. 135–154.

[50] Viarengo A, Nott JA (1993) Mechanisms of heavy metal cation homeostasis in marine invertebrates. Comp Biochem Physiol C 104: 355–372.

[51] Viarengo A, Ponzano E, Dondero F, Fabbri R (1997) A simple spectrophotometric method for metallothionein evaluation in marine organisms: an application to Mediterranean and Antarctic molluscs. Mar Environ Res 44: 69–84.

[52] Carajaville MP, Bebianno MJ, Blasco J, Porte C, Sarasquete C, et al (2000) The use of biomarkers to assess the impact of pollution in coastal environments of the Iberian Peninsula: a practical approach. Sci Total Environ 247: 295–311. 10803557 10.1016/s0048-9697(99)00499-4

[53] Cosson RP (2000) Bivalve metallothionein as a biomarker of aquatic ecosystem pollution by trace metals: limits and perspectives. Cell Mol Biol 46: 295–309. 10774921

[54] Isani G, Andreani G, Kindt M, Carpenè E (2000) Metallothioneins (MTs) in marine molluscs. Cell Mol Biol 46: 311–330. 10774922

[55] Palmiter RD (1994) Regulation of metallothionein genes by heavy metals appears to be mediated by a zinc-sensitive inhibitor that interacts with a constitutively active transcription factor MTF-1. Proc Natl Acad Sci USA 91: 1219–1223. 8108390 10.1073/pnas.91.4.1219 PMC43128

[56] Hamer DH (1986) Metallothioneins. Ann Rev Biochem 55: 913–951. 3527054 10.1146/annurev.bi.55.070186.004405

[57] Stuart GW, Searle PF, Chen HY, Brinster RL, Palmiter RD (1984) A 12-base-pair DNA motif that is repeated several times in metallothionein gene promoters confers metal regulation to a heterologous gene. Proc Natl Acad Sci USA 81: 7318–7322. 6095286 10.1073/pnas.81.23.7318 PMC392137

[58] Stuart GW, Searle PF, Palmiter RD (1985) Identification of multiple metal regulatory elements in mouse metallothionein-I promoter by assaying synthetic sequences. Nature 317: 828–831. 4058587 10.1038/317828a0

[59] Dalton TP, Palmiter RD, Andrews GK (1994) Transcriptional induction of the mouse metallothionein-I gene in hydrogen peroxide-treated Hepa cells involve a composite major late transcription factor/antioxidant response element and metal response promoter elements. Nucleic Acids Res 22: 5016–5023. 7800494 10.1093/nar/22.23.5016 PMC523772

[60] Dalton TP, Paria BC, Fernando LP, Huet-Hudson YM, Dey SK, et al (1997) Activation of the chicken metallothionein promoter by metals and oxidative stress in cultured cells and transgenic mice. Comp Biochem Physiol B 116: 75–86. 9080664 10.1016/s0305-0491(96)00224-6

[61] Haq F, Mahoney M, Koropatnick J (2003) Signaling events for metallothionein induction. Mutat Res 533: 211–226. 14643422 10.1016/j.mrfmmm.2003.07.014

[62] Siegel A, Sigel RKO (2009) Metal ions in life sciences. Vol. 5, RSC publishing, Cambridge, pp. 51–58. 10.1111/j.1748-3743.2009.00176.x 20925757

[63] Enshaei M, Khanafari A, Sepahey AA (2010) Metallothionein induction in two species of *Pseudomonas* exposed to cadmium and copper contamination. Iran J Environ Health 7, 287–298.

[64] Bourdineaud JP, Baudrimont M, Gonzalez P, Moreau JL (2006) Challenging the model for induction of metallothionein gene expression. Biochimie 88: 1787–1792. 16935407 10.1016/j.biochi.2006.07.021

